# A Meta-Analysis Review: Nanoparticles as a Gateway to Optimized Boiling Surfaces

**DOI:** 10.3390/nano14121012

**Published:** 2024-06-11

**Authors:** Giulia Motta, Antonis Sergis

**Affiliations:** The Department of Mechanical Engineering, Imperial College London, London SW7 2AZ, UK

**Keywords:** heat transfer, boiling, nanoparticles, efficiency, energy systems

## Abstract

Pool boiling is essential in many industrial manufacturing applications. In addition, it can become critical in the journey towards improving energy generation efficiency and accomplishing the goal of net-zero carbon emissions by 2050 via new or traditional power generation applications. The effectiveness of boiling is governed by the bubble cycle. The chemistry and topographical features of the surface being heated have been found to highly impact the boiling performance, such as in the case of pool boiling enhancement when employing hydrophilic and hydrophobic surfaces via nano/micro heater surface modification. Nevertheless, it is questionable how feasible it is to create these surfaces for large-scale applications due to their manufacturing and maintenance cost and complexity. The current work assesses whether the use of nanoparticles in traditional coolants could potentially unlock the mass production of optimised heating surface modification through a metadata literature review analysis. It was discovered that self-assembled layers created as a result of the deposition of nanoparticles in coolants undergoing pool boiling seem to behave most similarly to manufactured hydrophilic surfaces. The creation of enhanced patterned-heat transfer surfaces is shown to be possible via the use of a combination of different nanoparticle suspensions in coolants.

## 1. Introduction

The current metadata literature review analysis examines the improvement of boiling characteristics. Improving the upper limit of heat transfer from a given surface can potentially reduce maintenance costs [1], increase safety [2], and open the way for high-intensity energy applications that might help with future decarbonisation and energy efficiency (e.g., small modular nuclear reactors, nuclear fusion reactors etc. [3,4]). For all intents and purposes, optimising cooling systems is essential, as well is the improvement of pool boiling heat transfer along with a better understanding of its underlying physics.

Surface structure can strongly affect heat transfer [5]. In boiling heat transfer, this alters the shape of the Nukiyama curve [6] because surface morphology, such as roughness, influences nucleation sites by providing additional cavities where vapour is preferentially trapped and bubbles are allowed to grow and detach. Studies [7] have shown how different nanoparticle microporous surfaces can enhance boiling thermal performance thanks to changes in surface roughness and wettability, augmenting the critical heat flux up to 123% compared to that of a bare smooth copper surface. The effects of biphilic (mixed hydrophilic and hydrophobic) surfaces, as well as the auto-assembled surface patterns formed from suspensions of nanoparticles in liquids when boiled in nanoparticle suspensions, have shown promising results in enhancing the heat transfer performance of devices and processes. While biphilic surfaces can be manufactured to need, at large industrial scales, costs of production, maintenance and complexity limit the applicability of such surfaces. Therefore, alternative methods to create patterned surfaces that are cheaper, more efficient and easier to maintain are required, and the deposition of nanoparticles during boiling seems a promising solution. Research on nano-colloidal suspensions has attracted attention because of the observed superior thermal properties of heat transfer processes in the presence of nanoparticles [8,9]. The resulting morphology of the heated surface arising from the deposition of nanoparticles and the consequent effect on heat transfer performance is highly influenced by nanoparticle type, shape, size, and the concentration of the solution. Unfortunately, the physics and mechanisms of boiling, even for the simplest cases, are not fully understood, which makes optimization difficult. Many unresolved questions arise about the proposed new cooling techniques and the degree to which they might prove to be beneficial.

This paper aims to assess and evaluate previous experiments from the literature to suggest areas of expansion relevant to the field of boiling surface optimisation. It also attempts to explain the possible reasons for the existence of numerous discrepancies in pool boiling experiments employing suspensions of nanoparticles in coolants as working fluids. Starting from a review of pool boiling, this text will analyse previous studies on hydrophilic and hydrophobic surfaces, also evaluating the effects of their combined properties and the feasibility of adopting them in the energy industry. Pool boiling experiments using suspensions of nanoparticles in liquids are then reported and analysed, highlighting and investigating the possible reasons behind reported contradicting experimental results. A summary of the main nanoparticles used in the discussed studies is given in Table 1. The practicality of using colloidal suspensions will hence be assessed. Finally, future areas of research expansion on these aspects are suggested.

## 2. Materials and Methods

The research for the literature review was conducted according to the methodology outlined in Table 2.

The reason for neglecting studies involving surfactants is related to the fact that the objective of this literature research was to provide as much understanding as possible about the sole effects of such suspensions and biphilic surfaces. Surfactants and non-electrostatics stabilisers can age and be destroyed at elevated temperatures, which in turn could cause detrimental effects to suspension stability and the eventual thermal performance. Only one study that used a stabilizer is cited to show how it might have affected the results drawn. Moreover, it demonstrates, as many others have [19,20], how these stabilizers can hinder the transport properties. In contrast, it must be noted that other researchers [21] concluded that surfactants enhanced thermal performances. Thus, the uncertain nature of the effects, which for the majority were unfavourable to heat transfer, was another reason for neglecting in the analysis studies involving the use of surfactants and stabilizers. Secondly, nanoparticle characteristics in studies had to be explicitly stated, and where they were not, this was highlighted in the following manuscript, to avoid drawing conclusions from incompletely defined experimental conditions that can increase the probability of reaching ambiguous conclusions.

## 3. Background on Pool Boiling Heat Transfer and Its Thermophysical Applications

Heat management is generally important to system control and operational safety. Overheating due to inefficiencies in thermal management limits the durability of electronic devices, engines, power stations and many more applications, which are industrially, domestically and commercially important. Controlling heat flow can even become critical to facilitating future novel applications, such as nuclear fusion, as well as optimising current ones, e.g., improving thermal power plants. For boiling heat transfer, this can be accommodated via the optimisation of boiling surfaces. The design of optimised and high-energy-density cooling systems can follow from an understanding of the fundamental underlying physics that governs heat transfer processes. The current study focuses on pool boiling and applications that make use of this heat transfer mode. Although flow boiling is understandably extensively used (perhaps even at the same or greater levels as pool boiling) at industrial levels, the interaction of a flowing medium with bubble generation cycles and overall heat flux is an even more complex effect that has not been studied sufficiently.

Bubble dynamics during pool boiling affects two of the main characteristics of this phenomena: the heat transfer coefficient (HTC) and the critical heat flux (CHF). The introduction of instabilities augments the energy absorbed by the liquid, triggering bubble formation. In situations where substantial heat is absorbed in the absence of sufficient instabilities that can initiate the formation of bubbles, the onset of boiling (ONB) is delayed and occurs explosively thereafter (flash boiling) in the bulk of the liquid. Research in the field aims to find methods to enhance the thermophysical properties of boiling by improving the CHF, HTC and the ONB. Defects, such as cracks, are a form of instability, and act as sites for bubble generation: the contact area between the liquid and the heated surface increases and the trapped liquid readily vapourises. At nucleation sites, which additionally form at and around the location where bubbles are anchored before lift-off, energy is transferred in the form of latent heat from the liquid to the vapour phase.

### Contact Angles

Two parameters that influence bubble dynamics and boiling heat transfer are the “wettability” and the “contact angle” (Table 3). These parameters are going to be used later in the assessment and characterisation of the designed boiling surfaces, and are hence briefly discussed here.

The simplest method for measuring the contact angle is performed with the liquid droplet resting still on the surface [24], which defines the “static (or sessile drop) contact angle”. This has been found to be the most common type of measurement reported in the literature. Hence, to be consistent, in this work, when the term “contact angle” is used it will always refer to the static contact angle.

Thomas Young [25] established the laws for measuring the contact angle on chemically and microstructurally [23] homogeneous surfaces. These laws depend on the competing effects of liquid–vapour, solid–liquid and solid–vapour interfacial tensions [26], which eventually reach an equilibrium state where energy is at a minimum.

Nevertheless, surface defects and roughness alter the wetting behaviour of a surface. The extent of how much wetting occurs also depends on the external force required to overcome the surface’s resistance to liquid spreading, as the amount of energy in the region of the surface that has been wetted and the dry one around it will differ. When the energy of the surface under the liquid drop is lower than that of the dry area around it, the drop will preferentially spread, and when it is higher, the drop retains its spherical shape. When a surface is rough, more solid will be wetted due to the greater contact area, compared to a smooth one. Surface roughness and inhomogeneities therefore enhance the water-repelling or water-spreading features, and hence the Wenzel [27] and Cassie–Baxter [28] equations were developed [29] by modifying Young’s equation to take into account such surface inhomogeneities in homogeneous and heterogeneous wetting.

**Figure 1:**
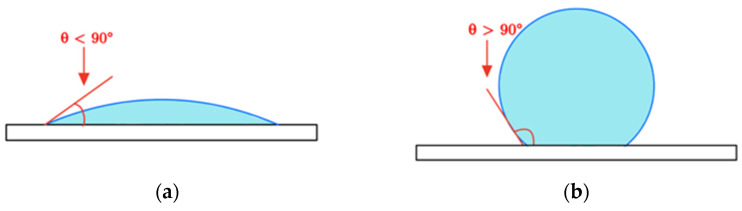
Droplet contact angles on hydrophilic and hydrophobic surfaces. (**a**) Water droplet on a hydrophilic surface; (**b**) water droplet on a hydrophobic surface.

A common use of the contact angle is to classify between two types of surfaces as hydrophilic (surface with a strong affinity to water, shown in Figure 1a) and hydrophobic (surface with a tendency to repel, as shown in Figure 1b). The basic characteristics of these surfaces are provided in Table 4 to aid the following discussion. The wetting capacity of a liquid onto a surface is mainly dependent on the interaction between intermolecular forces and the chemical characteristics of the surface [30]. Variations in surface microstructure and porosity instead influence the degree of hydrophilicity or hydrophobicity and its subsequent interaction with liquids containing polar molecules and ionic groups, such as water.

Gas bubbles in aqueous media show very similar behaviour to water droplets on a solid surface. For liquid droplets on a solid surface surrounded by a gas, the gas–solid phase around the droplet competes with the liquid–solid phase at the area of contact between the droplet and the solid. Similarly, on a solid surface immersed in a liquid, the gaseous bubble molecules compete with liquid ones adhering onto the solid surface [38]. Hence, the balance between liquid–solid–gaseous phases when droplets and bubbles are formed follows a similar process for gaseous bubbles in a liquid to that of liquid droplets in a gas. The contact angle is determined by the shape that a water droplet forms on a surface, which is in turn influenced by the surface tension of the fluid and the nature of the surface. Surface tension is an intrinsic property of fluids; thus for both droplets and vapour bubbles, it depends on the forces of attraction between liquid molecules and the fluid–solid interfaces surrounding the liquid.

## 4. Literature Results on the Effect of Surface Wettability on Pool Boiling

### 4.1. The Effects of Surface Wettability on the CHF, HTC and ONB

Surface wettability highly influences the CHF in pool boiling heat transfer. At the intermolecular level, the amount of wettability is also governed by the balance of adhesive forces, which cause the liquid to spread across the surface, and the cohesive forces within the liquid that resist the separation of molecules [25]. Cohesive forces act between molecules of the same substance, while adhesive forces determine the degree of attraction between molecules of different substances. Therefore, when cohesive forces overcome adhesive forces, the contact angle will be greater, whereas the opposite is true in the case where adhesive forces overcome cohesive forces.

Studies [25,39] have shown that hydrophilic surfaces enhance the CHF by ensuring a continuous supply of liquid to be evaporated, which delays surface dry-out. In contrast, hydrophobic surfaces tend to repel liquids from the surface and form vapour blankets more readily during pool boiling, which deteriorates the CHF [30,39,40] but also lowers the ONB, leading to a higher HTC.

Teodori et al. [30] focused on the effects of changing surface wettability solely through changes in surface chemistry. Bubbles nucleated in response to the alteration of the wall superheat of the tested surfaces placed on top of a heated copper cylinder during the pool boiling experiment of distilled water. As the copper cylinder was powered, heat transferred through the tested hydrophilic surfaces, which provided nucleation sites for phase change to occur. Bubbles of distilled water were observed to nucleate with the same average diameter on hydrophilic surfaces. As the heat flux was increased, bubble coalescence was minimal and delayed surface dry-out. In contrast, the departure diameter and the growth time of bubbles on a superhydrophobic surface increased with increasing heat flux, leading to early bubble coalescence and preventing effective heat transfer; this behaviour was attributed to the random micro-roughness of the surface.

Similarly, another experiment [41] involving a super-water-repellent surface (SWR), with a contact angle of 152°, observed a change in the typical pool boiling performance of water. The heated surface was a copper rod coated with a super-repellent layer formed from electrolytic nickel plating. At subcooling conditions, bubble nucleation started at a surface temperature below the saturation temperature. The same observation was reported by other researchers [42,43] and implied that the ONB began at a lower value of wall superheating than expected, enhancing the HTC. In this study, the phenomenon may be explained by the fact that when an embryonic (or starting/seed) bubble was formed in a hydrophobic cavity, the liquid pressure surrounding the bubble was greater than the vapour pressure of the bubble itself [40]. This implies that the saturation temperature of the vapour pressure of the bubble was lower than the liquid temperature. Therefore, the nucleating bubble, surrounded by superheated liquid, grew with no additional resistance. Once again, as commonly agreed upon by other studies [40,41,42], early bubble coalesce occurred because of a large contact diameter, leading to the Leidenfrost effect. Finally, the study suggested that the increasing number of active nucleation sites at low superheating values could develop more efficient heat transfer surfaces obtained by combining SWR coatings with super-hydrophilic surfaces.

Bubble dynamics represents one of the driving factors in boiling, and is a complex physical process that has yet to be fully understood. An investigation [44] into the growth mechanism of a single vapour-deionised (DI) water bubble in an artificial cavity on a weakly wetted surface better explained the heat transfer features previously described. The results obtained by an IR camera reveal that bubbles departing at any given heat flux left a small vapour substrate, which acted as a nucleation site for the next bubble. The ebullition cycle was only constituted by growth time, and it did not exhibit any waiting time because as soon as the bubble formed, it detached from the surface. The necking phenomenon at the bubble’s departure was identified as the cause of the vapour residue attached to the surface, which expanded uniformly in all directions, maintaining a receding contact angle of 90°.

Surface features are also closely interlinked with wettability and heat transfer performance. Differentiating between the effects of surface chemistry, porosity and roughness on the degree of wettability and the consequent impact on boiling performance is complex. For example, in a previous experiment [45], researchers changed the wettability of a copper surface, with an average surface roughness of 0.02 µm, through oxidation in air, which altered its porosity. The pool boiling experiment used saturated water at atmospheric pressure and heated the test surface using electrical heaters. Solving the one-dimensional heat equation using the data obtained revealed an enhancement in the CHF of water. The augmented boiling performance was a result of the change in porosity, which increased surface wettability by reducing the contact angle on two different surfaces from 90° to 18° and from 90° to 35°. The enhanced wettability was purely attributed to the alteration in surface characteristics. The surface chemistry of the heated surface alone did not have a large effect on the CHF. Decreasing the contact angle had a negligible effect on the average HTC: the number of bubbles nucleating in some artificial cavities on the surface substantially decreased with only a slight reduction in the HTC.

O’Hanley et al. [39] agreed that altering the surface wettability by changing the porosity of surfaces has a significant effect on the CHF. In their study, for some porous hydrophobic surfaces, the CHF of DI water was reduced by 96%, whereas for some smooth porous hydrophilic surfaces the CHF was increased by 60%. The CHF’s enhancement and degradation were only influenced by the wettability of the porous surfaces: the hydrophobic porous layer acted to repel even more water, accelerating the CHF, whereas hydrophilic pores increased the rewetting ability and delayed surface dry-out. Moreover, no influence on the CHF was observed when the roughness of the surfaces, ranging from less than 0.01 μm to 2.69 μm, was altered. The CHF was enhanced by 60% only for a combination of an average surface roughness above 7 μm and a high contact angle.

In contrast, other researchers [40] observed that an increase in microscale surface roughness further increased the contact angle of hydrophobic surfaces and lowered the bubble emission frequency. They explained that the higher contact area resulted in an augmented surface force, requiring a higher buoyant force to overcome it. This finding was similar to that of Phan et al. [42], who noted a strong dependence of the boiling performance of water on wettability. In their study, the surface roughness at the nanoscale was altered to obtain different degrees of surface wettability with static contact angles ranging from 22° to 112°. The bubble departure size increased with increasing the surface wettability of slightly hydrophilic surfaces (contact angles between 45° and 90°) and bubble emission frequency was reduced. The inverse effect was observed for very hydrophilic surface (contact angles of less than 45°): with increased wettability, the HTC improved because of a higher bubble emission frequency.

### 4.2. Summary of Findings for Pool Boiling of Hydrophilic and Hydrophobic Surfaces

Overall, the different effects encountered in experiments involving hydrophilic and hydrophobic surfaces on bubble dynamics are summarised in Table 5.

From the studies previously analysed, it emerges that boiling performance is mainly impacted by changes in wettability by varying surface chemistry and porosity. Moreover, while in some cases it also appeared that surface roughness affects the boiling performance, in others, it had a negligible effect. Hence, changes in the μ/nanoscale surface roughness of hydrophilic and hydrophobic surfaces must be further investigated, and repeated experiments with the same boundary conditions are required.

### 4.3. Pool Boiling Enhancement through Mixed Hydrophobic and Hydrophilic (Biphilic) Surfaces

Enhancing pool boiling is essential to achieving a better cooling performance. The boiling characteristics of hydrophobic and hydrophilic surfaces can be combined to form complex biphilic surfaces that could both enhance the CHF and the HTC and lower the ONB. Hydrophilic surfaces may help to delay surface dry-out by supplying liquid, aiding the rewetting of hydrophobic areas. Moreover, factors such as increasing the number of nucleation sites, decreasing the diameter of departing bubbles and bubble interactions can also potentially be controlled.

A careful choice of the geometry and manufacturing of the patterned surfaces may maximise boiling performance by controlling bubble dynamics. However, researchers do not currently have a clear understanding of the trivial physics giving rise to bubble dynamics phenomena and how these are affected when the heating surface morphology changes. Therefore, the optimal surface configuration of such complex patterned surfaces remains an open question. The efforts made in attempting to understand such effects are reported in this section.

A study by Sun et al. [46] used a cylindrical tank to conduct a pool boiling experiment with a patterned indium–tin oxide surface layered with PTFE (having hydrophobic characteristics and a contact angle of about 120°) and silicon oxide (having hydrophilic characteristics and a contact angle of 40°), using DI water as a working fluid. Purely hydrophobic and hydrophilic surfaces were also tested for comparison. As expected, the purely hydrophobic surface exhibited an early ONB and quickly reached the CHF. On the hydrophilic surface, bubbles grew individually with a small contact diameter and delayed surface dry-out. On the patterned surface, the combination of hydrophobic and hydrophilic surfaces led to a 90% increase in the CHF compared to that of a purely hydrophilic surface, and augmented the HTC.

Further, it has been reported by Betz et al. [47] that hydrophobic islands on a hydrophilic base surface (hydrophilic networks) have better thermophysical properties than hydrophilic islands on a hydrophobic base surface (hydrophobic networks). In both cases, the patterned surfaces showed superior pool boiling performance compared to homogeneous hydrophilic and hydrophobic surfaces. Nevertheless, while hydrophilic networks enhanced both the CHF and the HTC, hydrophobic ones only enhanced the HTC and, in some cases, led to a degradation in the CHF due to the large hydrophobic areas augmenting bubble coalescence. It was noted that to inhibit vapour blanketing effects and moderate instabilities, the distance between nucleation sites should be controlled by optimizing the spacing between hydrophobic areas.

The previous ideas were investigated in an experiment [48] using double-distilled water as a working fluid, testing different sizes, pitches, and densities of hydrophobic spots on a super-hydrophilic surface. The results were compared to those of a plain stainless-steel heater and a uniform superhydrophobic surface. The most promising result in the study was the enhancement of 61% of the average HTC and 200% of the CHF. A more detailed description of the configuration of the different tested patterned surfaces and associated performances is provided in Table 6.

The results confirm that the ratio of hydrophobic to hydrophilic areas, their geometry, and their distribution on a surface affects the boiling performance, especially bubble dynamics, and that smaller hydrophobic spots inhibit the Leidenfrost effect.

Furthermore, research by Lim et al. [49] devoted particular attention to factors influencing the bubble diameter and location on biphilic (patterned hydrophilic–hydrophobic) surfaces. Five biphilic surfaces were fabricated by coating porous superhydrophobic materials on a SiO_2_/Si hydrophilic bare heater with different sizes and pitches. A hydrophobic square surface with a contact angle of 150.5° was created by spray-coating and a uniform hydrophilic SiO_2_ surface with a contact angle of 41.8° was tested for comparison. The pool boiling experiment used DI water as a working fluid and concluded that biphilic surfaces with a smaller pattern size and pitch performed better than their base SiO_2_ surface, having higher CHF because of smaller bubble departure diameters. Hydrophobic sites provided additional nucleation sites, and their number was directly proportional to the HTC.

Studies [46,48,49] also describe that bubble generation on patterned surfaces seems to preferentially occur on the boundaries of hydrophobic and hydrophilic areas. The bubbles are then pushed onto hydrophobic islands by a net force arising from the unbalanced contact angles and are confined there until they detach, leaving a small vapour residue that facilitates the formation of the next bubble [49,50].

Sujith Kumar et al. [51] quantified the optimal size and pitch distance of hydrophobic islands created using polymethyl methacrylate resin and trichlorosilane as a hydrophobic agent. They designed square patterns on various cylindrical copper rods, with sizes varying from 1 mm to 3 mm and pitches from 4.5 mm to 5.5 mm, all in increments of 0.5 mm. The biphilic surfaces were compared to plain copper as a reference in a pool boiling experiment using DI water. At low heat fluxes, single bubbles developing on the boundary of hydrophobic regions were characterised by a long departure time (process from nucleation to departure). Bubble coalescence was more readily observed inside the same hydrophobic area and between different ones at higher heat fluxes. The pitch distance influenced how much coalescence occurred between hydrophobic islands. A smaller side-to-side distance between patterns resulted in a higher chance of bubble coalescence in the high-heat-flux regime. Even if all surfaces in the study demonstrated enhanced pool boiling, the surface with a pattern size of 2 mm and a pitch of 5 mm produced more nucleation sites and gave the lowest value of wall superheat. The study implied that the minimum inter-distance for optimal performance between hydrophobic areas should be around 3.2 mm at atmospheric pressure.

Jo et al. [50] further confirmed that pitch distance is one of the most significant parameters influencing the interaction phenomena of the bubbles. Contrary to the high heat flux regime, they found that in the low heat flux regime of tap water, a smaller pitch distance increased the density of bubbles, improving boiling heat transfer. Hence, an optimal balance of the side-to-side distance should be found to ensure sufficient and continuous surface rewetting. Similarly, the authors [50] also observed that the bigger the hydrophobic dot diameter of the pattern in the low-heat-flux regime, the better the boiling heat transfer and the earlier the ONB, whereas at high heat fluxes, a smaller diameter resulted in better heat transfer. Again, this was attributed to the fact that in the low-heat-flux regime, larger hydrophobic dots helped the creation of bubble nucleation sites; but with increasing heat fluxes, the bigger the hydrophobic areas, the more readily bubbles coalesce, forming a vapour film acting as a thermal insulator.

## 5. Analysis and Discussion the Optimization of Biphilic Surfaces

### 5.1. The Optimization of Biphilic Surfaces

All studies on biphilic surfaces have shown, in one way or another, superior thermophysical characteristics, compared to purely hydrophilic and hydrophobic surfaces. Moreover, an optimal balance between different geometrical parameters is critical in order to benefit from the effects of bubble dynamics at low and high heat fluxes, as, for example, bubble diameter seems to be directly related to the sizes of patterns.

The inter-distance between hydrophobic spots should be sufficient to keep the replenishing liquid reaching heating surfaces and prevent early-stage bubble coalescence. In previous studies, Sujith Kumar et al. [51] recommended values of around 3.2 mm for a particular set of experimental boundary conditions; however, generalisation to other applications should be further investigated. The pitch distances, sizes and shapes of hydrophobic areas on hydrophilic base surfaces are important and must be accurately determined and used to drive the design of biphilic surfaces.

It has been found that keeping the contact angle of the hydrophilic regions closer to 0° could potentially be beneficial to increasing the contrasting behaviour of hydrophilic and hydrophobic surfaces and improving their rewetting ability. To optimize biphilic surfaces in pool boiling heat transfer, surface topography, such as porosity, could also play a role. Further research should thus be carried out to confirm these findings and to assess the validity of the claims, and eventually understand and determine the optimal degree of porosity that could be used on a hydrophilic surface to enhance pool boiling.

Generally, the results suggest that hydrophobic islands should be manufactured on a hydrophilic porous base, and the optimal area ratio needs to be found to exploit the different benefits each type of surface has at low and high heat fluxes. One study by Motezakker et al. [52] sought to investigate this aspect, and found that the optimum ratio of hydrophobic surface to the total area of biphilic surface was 38.46%, which enhanced the CHF of DI water by 103%. However, any higher percentage of hydrophobic area resulted in an early formation of the vapour blanket. It is important to understand the physics that give rise to the limitations so as to develop designs for specific applications.

### 5.2. Feasibility of Using Biphilic Surfaces in Industrial, Commercial and Domestic Applications

It is important, along with an evaluation of their thermophysical properties, to assess how practical it is to implement biphilic surfaces at a large scale. It is essential to determine if their often complex and currently expensive manufacturing processes are worth the benefits of their displayed enhanced heat transfer properties. There is also a need to evaluate the practicality of making biphilic surfaces and applying them to large-scale industrial applications, and to assess their longevity and the associated maintenance and/or re-generation efforts (and cost) that are required to keep them functioning. Some of the techniques employed to manufacture hydrophilic and hydrophobic surfaces are summarised in Table 7.

Many of the manufacturing processes to produce hydrophilic surfaces are expensive and involve toxic substances. In addition, the manufacturing of hydrophobic surfaces often involves numerous steps, making the techniques complex and time-consuming, which will impact manufacturing cost.

Scalable manufacturing methods for the production of biphilic surfaces have been investigated [42], but there is little to no information with regard to biphilic surface longevity in a real, mass produced device for purposes of industrial boiling heat transfer. There have been attempts [57] to fabricate superhydrophobic surfaces using simple coating methods, such as by the grafting of fatty acid molecules onto aluminium surfaces, which have been demonstrated to be able to withstand exposure to hostile conditions. Nevertheless, in industrial applications, various factors, such as heat, acidic environments, etc., affect the boiling surface all at once, and it is therefore difficult to assess the durability of surfaces using small-scale laboratory experiments.

One of the potential applications where biphilic surfaces could be of use is thermal power plants. These require large water cooling systems [58], and the use of biphilic surfaces may improve their efficiency and in effect increase their energy density. The maintenance of cooling systems is a major concern because of scale deposition, corrosion, fouling [58] and abrasion, which may influence the desired performance of biphilic surfaces over time if potentially used in such systems. Industrial boilers often employ invasive cleaning techniques such as the use of explosives and shock pulse water guns to break down the deposited layers of minerals. Hence, the delicate biphilic surfaces and their specific geometry could be destroyed in the process of scale removal. The repair/regeneration of biphilic surfaces in such systems is expected to be practically difficult and costly or/and even impossible. Studies by Ferrari et al. [59] have indicated the use of hydrophobic surfaces to prevent mineral deposition and fouling, exploiting their self-cleaning property. It is not yet clear though how this property could be applied in large industrial systems, or what would be the effect on surfaces with a combination of hydrophobic and hydrophilic areas. Therefore, the usability of biphilic surfaces has so far been limited, making their production unappealing, especially for large-scale applications where cost and safety are paramount.

## 6. Results on the Effect of Nanoparticles on Pool Boiling

### 6.1. Overview of Nano Colloids for Thermal Applications

Nano colloids (nanoparticle suspensions) are mixtures of a base fluid and nanosized particles with an average size of less than 100 nm. These substances have been of particular interest since the year 2000 because of their potentially enhanced heat transfer properties. Nanoparticle suspensions were first proposed by Choi in 1995, who aimed to overcome the low thermal conductivity of liquids, which he defined to be “a primary limitation in the development of energy-efficient heat transfer fluids that are required in many industrial applications” [60]. The surface to volume ratio of nanoparticles was believed to enable more solid to liquid and solid to solid molecular surface interactions, benefiting from energy transfer in the form of heat. This was originally attributed to the increased heat transfer conductivity of nanoparticles, which could in turn increase the mixture’s overall thermal performance. However, the physics of heat transfer in such nano colloids has and continues to be a source of controversy and dispute amongst researchers. In the absence of fundamental understanding and standardisation in their preparation methods, and subsequently adequately bound experimentation, they have remained underutilised since the discovery of their thermal effects.

Nano colloids are classified based on the nanoparticles they contain as metal-based, metal oxide-based, carbon-based and hybrid/mixed metal-based. Most studies have investigated the performance of the metal oxide-based types because of their lower cost compared to others [61]. Unfortunately, results regarding the impacts of nanoparticle suspensions on pool boiling heat transfer are often contradictory due to the complex nature of these substances. Previous literature has identified both degradation and enhancement, reporting in some cases a minimum higher heat transfer of 30% to 60% during nucleate boiling [62], and in others, a minimum reduction up to 55% [63].

One of the main issues in nanoparticle suspensions undergoing heating and cooling cycles is that nanoparticles tend to agglomerate, influencing the thermophysical properties of their base fluid. This phenomenon is reported to potentially be a consequence of the increased Brownian motion and a higher probability of collisions of particles during heating, which leads to the formation of clusters [61], making the mixture less stable. The stability of colloidal suspensions, such as nanoparticle suspensions, is understood through the Derjaguin, Landau, Vewey, and Overbeek theory [64], whereby the total potential energy of the particles is the sum of the total van der Waals attractive potential and electrostatic repulsive potential. When the attractive potential dominates over the electrostatic repulsive potential, the nanoparticles agglomerate together [61] and are then deposited by the action of gravitational forces [65]. In cooling systems, unstable colloids may lead to additional resistance to heat transfer in the form of fouling.

There are several techniques that have been applied to improve the stability of nanoparticle suspensions; for example, surfactants have been widely used. Nevertheless, surfactants tend to degrade at higher temperatures and can have catastrophic consequences on the stability of nanoparticle suspensions [61]. In the current work, nearly all articles involving surfactants have been neglected because of their potential influence on the heat transfer performance of nanoparticle suspensions, which might erroneously skew understanding. Surfactants have a hydrophobic tail and a hydrophilic polar head group resulting in a higher wettability [66]. Therefore, it is very likely that these substances may interact with and influence nanoparticle suspensions in pool boiling by affecting the viscosity and thermal conductivity, hindering heat transfer in a non-repeatable or/and hysteretic way [20].

### 6.2. Pool Boiling of Nano Colloids

Extensive research has been carried out to assess the impact of nanoparticle suspensions on heat transfer and pool boiling, but results are still unclear. There are several obstacles to the study of pool boiling itself, as the general behaviour of bubbles and the HTC are strongly dependent on the specific test conditions and system features (e.g., the heater geometry, the heating surface properties [67], etc.). Nevertheless, it seems that the main external factors influencing the boiling performance, such as the concentration of the working fluid, the topography of the heated surface and the wettability, are all interlinked.

Kouloulias et al. [10] studied the pool boiling performance at atmospheric conditions of DI water compared to a diluted Al_2_O_3_–H_2_O nanoparticle suspension, using a Ni–Cr wire as the heating element. Two different nanoparticle suspensions of concentrations 0.0012 vol% and 0.0024 vol% with a spherical shape and average diameter of 50 nm were tested [68]. Overall, a similar boiling curve to that of water was observed. Differently from water, as the heat flux was increased, more nucleation sites became active and the bubbles grew larger in size. The authors explained that the presence of nanoparticles led to extended bubble coalescence. Furthermore, the study reported, similarly to others [11], that increasing the nanoparticle concentration from 0.0012 vol% to 0.0024 vol% had no significant effect on boiling performance.

Other experiments [10,63,69,70,71] observed a change in the typical boiling performance of water when using nanoparticle suspensions, attributed to the deposition of nanoparticles on the heated surface forming a porous layer. The deposited porous layer was found in some cases [69,70] to enhance the HTC until a certain concentration of nanoparticles, and then degrade it. At low concentrations, a small amount of deposition led to an increased bubble emission frequency by providing additional nucleation sites [69,72]. In contrast, for larger nanoparticle concentrations, the deposition increased substantially, resulting in additional resistance to heat transfer.

**Figure 2:**
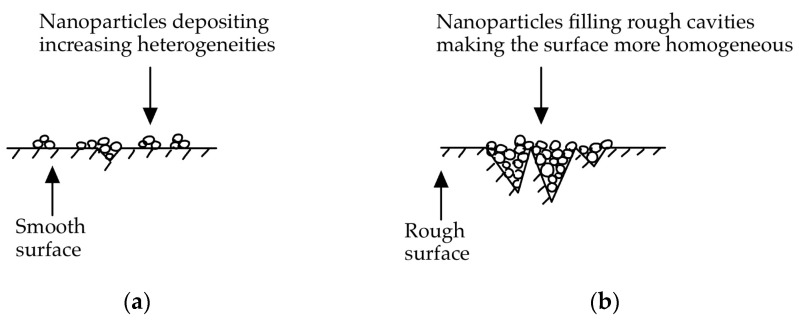
Nanoparticle deposition on different surfaces’ microstructures. (**a**) Deposition of nanoparticles on a smooth surface. (**b**) Deposition of nanoparticles on a rough surface.

Similarly, a different study [12] also attributed changes in boiling performance using nano colloids to alterations in surface topography, but this time to microscale surface roughness and not porosity. The working fluid used was a water-based γ-Fe_2_O_3_ nanoparticle suspensions with an average particle size of 10 nm and with no well-defined particle shape stated in the experiment. The authors explained that on a smooth heater surface, small particle deposition increased the number of nucleation sites due to an increase in surface roughness (Figure 2a). On the contrary, with an increase in nanoparticle concentration, when starting with a surface already characterised by microscale roughness, heat transfer resistance in the form of fouling occurred; the bubble nucleation sites were filled with nanoparticles, as shown in Figure 2b, making them no longer active and diminishing the nucleation site density.

As well as the HTC, the CHF is also affected by particle concentration. Kim et al. [13] investigated the change in CHF, aiming to understand the importance of nanoparticle deposition in heat transfer enhancement. Three experiments used water-based nanoparticle suspensions with five different volume concentrations ranging from 10^−5^ vol% to 10^−1^ vol% (one containing polygonal TiO_2_ nanoparticles and the other spherical Al_2_O_3_, with a mean particle diameter of 23 nm and 47 nm, respectively) on a bare Ni–Cr heater. The first experiment involved boiling pure water on the bare heater to function as the reference scenario. In the second experiment, the different particle-type nanofluids with different concentrations were boiled in the presence of the bare heater. In the third experiment, water was boiled in the presence of the nanoparticle-coated heaters, obtained from a boiling experiment similar to the second experiment. The results of the experiment are reported in Table 8.

The nanoparticle deposition on the bare heater after the experiment was mainly attributed to the nucleation of vapour bubbles, not gravitational effects, as it was impossible to recognise differences between the top and bottom sides of the wire. The study concluded that the CHF enhancement of pure water on the nanoparticle-coated heater was solely attributable to the change in surface characteristics, such as wettability, caused by the nanoparticle coating. The differences in the observed enhancements between the two nanofluids could be attributed to a series of factors, including particle type, size, shape and concentration, but to understand the main driver, a further investigation to decouple these effects should be conducted.

A CHF increase was also reported in Bang and Heung Chang’s [14] experiment, where the improved the boiling performance of the CHF in a horizontal test section by about 32% and in a vertical one by 13%. Alumina nanoparticles with a spherical shape and size ranging between 10 nm and 100 nm, suspended in water, were used. While the CHF was enhanced, the HTC was lower than that of pure water because of a reduction in active nucleation sites and a longer natural convection regime. The authors attributed their observations to changes in surface roughness caused by nanoparticle deposition, once again fouling the system, which initially had a surface roughness smaller than the size of the nanoparticles. To a certain extent, it was argued that the reduction in active nucleation sites was beneficial to the CHF by decreasing bubble coalescence and the formation of large vapour blankets.

Mixtures of different chemical element nanoparticles in base fluids (hybrid nanofluids) have also been investigated, with efforts to identify the optimal combination of properties to enhance thermal performance. In particular, one study [18] compared the performance of hybrid and monoparticle nanofluids containing boron nanoparticles of 7 nm and silica nanoparticles of 20 nm, with all solutions having a volume concentration of 0.05 vol%. The hybrid nanoparticle solutions contained different proportions of boron and silica: 0.01/0.04, 0.02/0.03, 0.03/0.02, and 0.04/0.01 (Boron vol%/SiO_2_ vol%). The base fluid consisted of a mixture of ethylene glycol and distilled water. The pool boiling experiment used a Ni–Cr wire as a heating element. The results confirm once again an enhanced thermal performance (better HTC and higher CHF) of the noncolloidal suspensions compared to the base fluid solution when undergoing pool boiling. It was noted that the HTC augmented with increasing boron concentration in the hybrid nanofluid solution, achieving a maximum enhancement of 52%. As concerns the CHF, both the monoparticle and hydrid nanoparticle solutions increased the CHF compared to the base fluid, and it was observed that increasing the boron concentration of the hybrid solution degraded the CHF. The two most significant enhancements in CHF where registered to be 69% for BN/SiO_2_ of 0.04/0.01 and 51% for 0.01/0.04. The efforts of this study suggest that different combinations of chemistries of nanoparticles could also be optimised to improve boiling performance. The observed enhancements were attributed to the deposition of nanoparticles on the surface increasing the amounts of pits and the heterogeneity of the surface, augmenting the number of bubble nucleation sites.

It is generally believed that nanoparticle deposition on heated surfaces is the reason for the variation in boiling performance with nano colloids. If the deposition of nanoparticles was controlled to act in a way that could alter the wettability of a heated surface and therefore bubble dynamics, this may be controlled to obtain the desired boiling performance (similarly to hydrophobic/hydrophilic/biphilic surfaces). In fact, in an experiment [15] involving aluminium oxide (particle size ranging from 110 nm to 210 nm), zirconium dioxide (particle size ranging from 110 nm to 250 nm), and silicon dioxide (particle size ranging from 20 nm to 40 nm) for nanoparticles with no specified shape, suspended in water, it was clearly observed that soon after boiling was initiated, some nanoparticles precipitated on the heater’s surface. Once again, irregular porous structures were formed and the heater’s surface contact angle reduced from 70° to 20°. The increased wettability explained the CHF enhancement typical of hydrophilic surfaces: the deposited nanolayer increased the capacity for rewetting of dry patches on the surface, preventing early surface superheating.

In contrast, it was also found that during pool boiling with an alumina–water nanoparticle suspension [11] with a concentration of 49.5 wt. %, the CHF degraded significantly by 22% on an oxidised copper heater (a surface with high wettability) and by 13% on an unoxidized copper heater (a surface with low wettability). Only with increasing nanoparticle concentrations, up to twenty times the initial value, was the previously reported CHF enhancement observed. It should be noted, though, that in this experiment, the nanoparticles were suspended using a dispersant agent. It is possible that the stabiliser used affected the results, and this would explain the disagreement with previously reported studies. Moreover, the size of the nanoparticles was stated to be 10 nm, as specified by the manufacturer, with no further investigation, and no information about their shape was provided.

Quan, Wang and Cheng’s study [16] is one of the few wherein the behaviour of nanoparticles in pool boiling was studied through a detailed visualisation of bubble dynamics and the inspection of the deposited nanoparticle layer on the heated surface. A two-step method was used for the preparation of the nanoparticle suspensions (having a particle concentration of 0.04% by weight and an average diameter of 25 nm). The surfaces of the silica nanoparticles, which were implied to have a spherical shape, were modified using different binary silicon–hydrogen compounds, making the nanoparticles in one of the fluids “strongly hydrophilic” and in another “moderately hydrophilic”. The study explicitly stated that no surfactants were used so as to avoid influencing the results. Three pool boiling experiments in a Pyrex glass container, using a copper heater, were carried out with these colloids. Each of the three heaters used in the experiment was polished with the same sandpaper properties to ensure the same surface roughness.

The enhancement or deterioration of the boiling performance (Table 9) of the two nanoparticle suspensions compared to their base fluid was attributed to the difference in wettability of the constituent nanoparticles.

**Figure 3:**
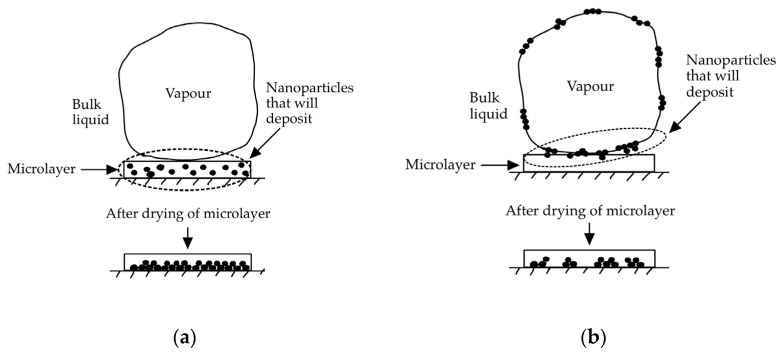
Nanoparticles’ interactions with bubbles on a (**a**) strongly hydrophilic heated surface and a (**b**) moderately hydrophilic heated surface.

Additionally, the enhancement and deterioration observed were further explained by the behaviours of nanoparticles, and how these interacted when bubbles were formed. Figure 3a,b show, respectively, how the strongly hydrophilic and moderately hydrophilic nanoparticles were distributed and deposited on the heater’s surface.

The large agglomerates formed from moderately hydrophilic nanoparticles increased the surface roughness by 817 nm, while, for the highly hydrophilic nanoparticle suspension, the deposited particles increased the surface roughness only by 66 nm, because of their much more uniform deposition. The larger nanoscale surface roughness was the cause of an increased boiling performance in the presence of hydrophilic nanoparticles: the number of nucleation sites increased, enhancing the hydrophilic properties of the heater, therefore delaying the critical CHF.

## 7. Discussion of the Literature on the Effects of Nanocollidal Suspensions on Boiling Heat Transfer

### 7.1. Contradicting Literature Findings

The scarcity of agreement between studies involving nano colloids renders it unclear which factors influence their behaviour during pool-boiling, which is essential to making progress in the field.

The conclusions reported in the previous section were mainly derived from pure observation, usually with poorly defined boundary conditions and scarce explanations about the underlying physics involved. As such, the lack of physical description in the findings and the contradicting results make it hard to determine how and why nanoparticle suspensions might or might not benefit cooling systems. The contradicting studies about the performance of nanoparticle suspensions may be additionally attributed to a lack of a standardised procedure and fixed experimental parameters. Moreover, neglecting articles involving the use of surfactants and stabilizers narrowed the amount of literature available, helping us to avoid biased conclusions.

Furthermore, explanations of the preparation processes of nanoparticle suspension solutions in the various experiments were frequently found to be insufficient/not detailed enough. Often, researchers had complete trust in the nanoparticle suspensions/nanoparticles purchased from suppliers without adequate confirmation and characterisation of the purchased products or their final prepared mixtures. This does not imply that the working fluids were not suitable for the experiments, but the absence of an exhaustive amount of description of the particles’ shapes and size, and techniques used, may lead to unclarity and difficulty in assessing the validity of such findings. This has been made evident in the absence of consensus in the findings from similar and identical experiments documented in the open literature.

In various articles, it seems that nanoparticle deposition and the wetting characteristics of the deposited surfaces are the driving factors to which the enhancement or degradation of boiling performance is attributed—this is a result that has found general consensus in the literature. Nevertheless, a clear explanation as to why and how nanoparticles deposit the way they do has not been provided. This is an essential task that will guide the design process for such systems.

### 7.2. Feasibility of Potential Auto-Generated Biphilic Surfaces in Industrial, Commercial and Domestic Applications

The different studies examined suggest that the enhanced boiling performance of nano colloids is driven by a self-assembled nanoparticle layer, which deposits on the surface. The reported enhancement of the CHF was associated by most studies with the porous nature of this deposited layer. If this is confirmed, understanding and controlling the nanoparticle deposition and agglomeration of boiling surfaces could be vital, affecting the way cooling systems (new or retrofitted) are designed and optimised to take advantage of these heat transfer-enhancing effects.

As previously reported [49], it was found that larger hydrophobic islands on hydrophilic-based surfaces perform better in the low-flux regime, while smaller ones perform better in the high-flux regime. Potentially, the deposited layers could also be dynamically created and controlled by altering the concentration and types of nanoparticles used in coolants, leading to online and in situ regeneration, altering the heat transfer surfaces as needed. An interesting way of developing this finding along with a better understanding of nanoparticle agglomeration would be to find a way to dynamically control the deposition of nanoparticles in such a way that the hydrophilic and hydrophobic islands could be optimised or dynamically altered on an application-specific basis.

An experiment [17] already attempted to obtain different microstructures by controlling the deposition of a water-based titania (TiO_2_) nanoparticle suspension with a concentration of 0.01 vol%. An SEM image was taken of the dry TiO_2_ nanoparticles. As the heat flux rate was increased, the boiling phenomena were observed to become more vigorous, and there was intensified nanoparticle deposition resulting in a thicker and rougher surface microstructure. A slower rate of heat flux increase for the same maximum values resulted in more coarse deposits, with an intensified aggregation of nanoparticles on the surface and larger resulting microstructures. The modified microstructures resulted in an increased wettability with a contact angle reduction on the bare Ni–Cr wire, from about 70° to a range between 10° and 20° for the different deposited surfaces.

Das, Putra and Roetzel [67] have shown that the nanoparticle suspensions used for pool boiling on rough surfaces cause degradation in boiling characteristics directly proportional to the concentration of the nanoparticle suspension. The study attributed the lower thermophysical performance of nanoparticle suspensions to the deposition of the nanoparticles decreasing the surface roughness. This could imply that surfaces featuring nanoparticle suspensions should initially be as smooth as possible in order to benefit from particle deposition [61] that avoids degrading the boiling performance. The selective chemical composition of nanoparticles and their deposition to create hydrophobic and hydrophilic regions in industrial boilers early after a cleaning cycle could reduce the amount of maintenance required in such systems. The ability to dissolve, recreate and alter the auto-assembled biphilic surfaces on demand is thus enticing.

## 8. Concluding Remarks and Highlighting Future Research Needs

The aim of the review was to quantify and understand the boiling heat transfer enhancement observed in traditionally manufactured nano/micro-textured heat transfer surfaces, as well as those with surface coatings automatically formed when boiling liquids (nano colloids) latent in nanoparticles. The effort focused on identifying and comparing the boiling dynamics leading to heat transfer enhancement for both types of surface assembly considered (i.e., traditionally manufactured and auto-assembled), and gauging their potential for large-scale deployment. The most impressive CHF enhancements in boiling reported in the literature were 200% for traditionally manufactured biphilic surfaces and 176% for nano colloids.

The analyses of data from the literature on hydrophilic/hydrophobic surfaces, nano colloids and pool boiling suggest that the self-assembled nanoparticle layer has a strong affinity to traditionally manufactured hydrophilic features due to the similarity of its distinctive porous structure and observed CHF enhancement characteristics. Further relevant and appropriately bound investigations are needed to advance the understanding of the novel auto-assembled nanolayers observed in boiling nanoparticle suspensions, which could eventually lead to their assembly control. This field is still immature, as made evident through the many conflicting observations scattered throughout the literature pertaining to nanoparticle suspensions and their associated boiling heat transfer effects, which have been detailed in the current investigation. The current authors believe that these disparities could be addressed through the development of a protocol to standardise the manufacturing and experimentation (or even testing) of nanoparticle suspensions that clearly defines and applies controls on essential suspension parameters, such as nanoparticle shape, concentration and type, and experimental conditions. More fundamental experiments should thereafter be executed in a parametric manner to delimit the suspension control aspects related to the characteristics of the emerging auto-assembled layers and their associated heat transfer performance. An erosion study using such materials will also be required. Other issues that should be addressed in employing nanocolloidal suspensions at the industrial scale include the stability of nanofluids and the interaction of nanoparticles with system components such as pumps. As also recognised by other authors [61], the long-term stability and the reusability of nanofluids are key characteristics that will allow for efficient industrial use. Future research should address the topic by testing combinations of dielectric constants, the pH values of base fluids, the shape, type and size of particles, as well as zeta potential in the presence of industrial operating conditions. Secondly, if nanofluids were to be used in existing industrial applications, hydrophobic coating technologies should be explored to increase the durability of the component [73,74].

Contrary to traditionally manufactured biphilic surfaces, auto-assembled nano surfaces can be deployed and thereafter maintained easily and cost-effectively with existing and new components. These can allow for incorporating self-healing and dynamic control aspects into their design, hence offering unprecedented design and operational flexibility. Such heat-transfer-related surface modifications could enable the user to replicate the generation of optimal traditionally manufactured biphilic heat transfer surfaces using nanoparticle suspensions and a controlled nanoparticle deposition process. The new enhanced surfaces could potentially allow for the extension of the boiling heat flux safety margins, leading to new, higher-energy-density applications or/and improving those currently in use through retrofitting. These new high-density small-form-factor heat transfer systems could hence be used in a broad range of new applications, not only in the high-performance micro-electronics industry, but also, more interestingly, in electricity and heat production. For example, they could become essential in the deployment of high-energy-density decentralised power plants that could provide peak/baseline loads in a future renewables energy grid or/and allow the development of power production applications currently limited by their high heat fluxes; these could range from (but are not limited to) nuclear fusion, as well as small-modular and micro-nuclear fission reactors.

## Figures and Tables

**Table 1:** Nanoparticles used in discussed studies.

Study	Nano Particle Elemental Composition	Shape	Size
[10]	Al_2_O_3_	Spherical	50 nm
[11]	Al_2_O_3_	Not well-defined	45 nm
[12]	γ-Fe_2_O_3_	Not well-defined	10 nm
[13]	TiO_2_	Polygonal	23 nm
[13]	Al_2_O_3_	Spherical	47 nm
[14]	Al_2_O_3_	Spherical	10–100 nm
[15]	Al_2_O_3_	Not well-defined	110–220 nm
[15]	ZrO_2_	Not well-defined	110–250 nm
[15]	SiO_2_	Not well-defined	20–40 nm
[16]	SiO_2_	Spherical	25 nm
[17]	TiO_2_	Not well-defined	45.6 nm
[18]	B	Not well-defined	7 nm
[18]	SiO_2_	Not well-defined	20 nm

**Table 2:** Literature research methodology criteria.

Step 1: Resource identification	- Keywords including: “heat transfer”, “enhancement”, “degradation”, “pool boiling”, “boiling”, “nanoparticle suspensions”, “hydrophilic”, “hydrophobic” and “surface microstructure” were used to filter studies according to the topic of interest. - Research tools used included: Google Scholar and Engineering village to collect journal articles, books and reports on the subject of pool boiling involving, hydrophilic and hydrophobic surfaces and nanoparticle suspensions.
Step 2: Validation resources	- All articles were assessed on the website Scimago Journal and Country Ranks. - The Q factor was employed to identify the quality of different journals on the subject. Resources with a higher Q factor were preferred and conclusions were drawn from them. - Articles with similar experiments and investigations were instead used to confirm and verify the findings reported.
Step 3: Resource filtering	- The articles chosen were further restricted by two main criteria: (1) Experiments on nanoparticle suspensions using surfactants and non-electrostatic stabilisers during their preparation were neglected. (2) Exerimental boundary conditions, such as nanoparticle size concentration and shape, had to be well-defined and controlled in the experimental procedures.

**Table 3:** Description of wettability and contact angle.

Parameter	Description
Wettability	- Is the ability of a liquid to remain in contact with a solid surface.
Contact angle	- Quantifies the wettability of a surface by measuring the angle between the droplet’s edge made by the liquid of interest and the surface. - Measurements vary with the surface under study and are strongly dependent on how it is being measured; e.g., whether it is measured for liquid that advances or recedes, or when the droplet simply rests stationary on the surface. - The difference between the advancing and receding contact angles defines the contact angle hysteresis (CAH), which quantifies the adhesion of the liquid onto the surface [22,23].

**Table 4:** Characteristics of hydrophilic and hydrophilic surfaces.

	Hydrophilic Surfaces	Hydrophobic Surfaces
Characterisation by surface chemistry	- High surface energy and density of polar groups. - Water spreads on these surfaces containing electric dipoles (“polar spreads on polar” [31]).	- Non-polar. - Water meeting a hydrophobic smooth surface has a low degree of interaction through van der Waals forces [26].
Contact angle	- Have a contact angle less than 90°. - Super hydrophilic surfaces have a contact angle between 10° [32] and 0° [33]. - Exhibit complete wetting behaviour, hardly forming any water droplets.	- Have a contact angle greater than 90°. - Superhydrophobic surfaces have a contact angle greater than 150°. These are found in nature, like the lotus leaf, which exhibits a self-cleaning behaviour [23]. Superhydrophobic surfaces also require low hysteresis [34].
Effects of surface topography	- Surface roughness magnifies the degree of hydrophilicity: the net increase in surface contact area leads to a larger polar interaction.	- Surface microstructure can increase the degree of hydrophobicity [35,36,37]. - Increasing the surface roughness increases the hydrophobicity of a surface by augmenting the free energy at the solid–liquid interface.
Some applications	- Biomedical, filtration, heat pipes [23].	- Anti-corrosion, drag reduction, oil–water separation, anti-fogging [32].

**Table 5:** Summary of findings on hydrophilic and hydrophobic surfaces.

Surface	Bubble Dynamics in Pool Boiling Experiments
Hydrophilic	- High bubble emission frequency. - Smaller contact diameter and bubbles tend to grow without interfering with each other. - Enhance the CHF by improving the rewetting of hot spots, enhancing boiling performance at higher heat fluxes.
Hydrophobic	- Lower ONB. - Higher HTC at lower heat fluxes. - Small vapour residues are left by the departing bubble, easing bubble nucleation. - Bubbles have larger contact diameters, which, at high heat fluxes, promote bubble coalescence and lead to the Leidenfrost effect.

**Table 6:** Performances of the different types of patterned surfaces in the study [48].

Features	Surface 1	Surface 2	Surface 3
Area of hydrophobic regions (mm^2^)	2.0 × 2.0	1.0 × 1.0	0.25 × 0.25
Pitch (mm)	4	3	1.5
Hydrophobic spots density (cm^−2^)	6.3	11.1	44.4
HTC	On average 10% lower than Surface 3	Similar behaviour to Surface 1	On average a 10% higher HTC compared to Surface 1 at all heat fluxes, being 51.2 kW/m^2^
CHF	Virtually no difference to the uniform superhydrophobic surface tested for comparison. On average exhibited a 200% increase in CHF compared to a bare stainless-steel heater.		
Bubble dynamics and pool boiling features observed	- The bubble departure diameter was 1.7 mm - Vapour film was observed to form on hydrophobic spots	Experiment only stated it behaved like Surface 1	- The bubble departure diameter was 0.9 mm - Had highest density of active nucleating sites - No vapour film formation

**Table 7:** Methods of manufacturing hydrophilic and hydrophobic surfaces.

Technique	Description
Hydrophilic surfaces	
Deposition of molecular film [31]	- Organic molecules are absorbed onto the solid surface from a solution or in vapour form. - Hydrophilic surfaces are obtained if the end of the deposited layer is polar. - The technique sometimes suffers from poor stability.
Modification of substrate chemistry [31]	- The most widely used methods are: plasma, corona and flame treatments. - Aims to produce free radicals and highly oxidised surfaces are therefore obtained by the creation of polar groups. - A basic chemical method to augment the hydrophilicity of the substrate involves oxidation.
Hydrophobic surfaces	
Layer—by—layer assembly	- Four-step method. - Often requires aggressive surface pre-treatment.
Chemical etching [23]	- Requires five steps (cleaning, masking, scribing, etching, and demasking). - Used in the aerospace industry for large components making the process suitable for large scale applications. - Process can be applied only to metallic elements.
Hummers’ method [23]	- Chemical process to oxidize the surface. - Uses sulfuric acids (highly hazardous substances). - Designed specifically for graphite oxide and can be applied to a limited number of materials
Chemical or Physical Vapour Deposition	- Chemical vapour deposition involves a solid product depositing on a substrate through a gas phase or surface reaction [53]. - Physical vapour deposition employs a phase change reaction to form a thin film [54].
Coating methods	- These include dip-coating and drop-casting. - In dip-coating a film of coating material is applied on a substrate by submerging it in a liquid solution and then withdrawing it at a constant speed [55]. - In drop-casting, a drop of liquid with the particles suspended is deposited on the surface [56].

**Table 8:** Results comparing nanoparticle suspensions and heaters coated with nanoparticles [13].

Working Fluid	Heater Surface	Effect Compared to Reference Experiment
Experiment 2 using nanoparticle suspensions on bare heater surfaces		
Water–TiO_2_ nanoparticle suspension	Ni–Cr heater	CHF increased to 170% for particle concentrations up to 10^−3^ vol%, after which little change was observed
Water–Al_2_O_3_ nanoparticle suspension	Ni–Cr heater	CHF increased to 180% for particle concentrations up to 10^−2^ vol%, after which little increase was observed
Experiment 3 using heaters coated with nanoparticles and pure water as a working fluid		
Water	Heater coated with nanoparticles	The CHF enhancement of pure water on the nanoparticle-coated heater was like that of the nanoparticle suspension (for all particle concentrations) on a bare heater, within the range of experimental uncertainty

**Table 9:** Nanoparticle wettability, bubble dynamics and effect on heat transfer [16].

Nanoparticle Wettability	Bubble Dynamics	Effect on Heat Transfer
Moderately hydrophilic	Smaller bubbles in both the low- and high-heat-flux regime, leading to higher emission frequency.	High CHF and HTC in high-heat-flux regime.
Super hydrophilic	Larger bubble departure diameters and bubble coalescence were observed (similar to boiling water).	Lower CHF and nucleate boiling heat transfer rate to the base fluid.
